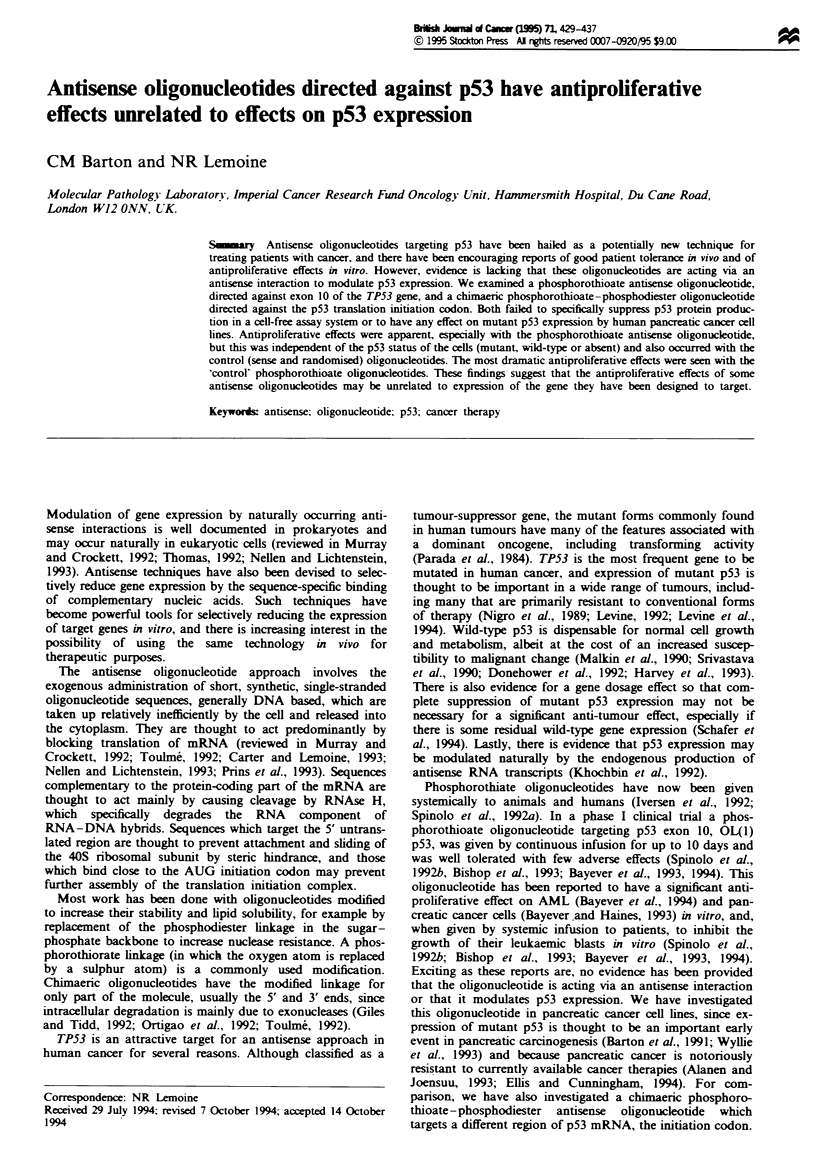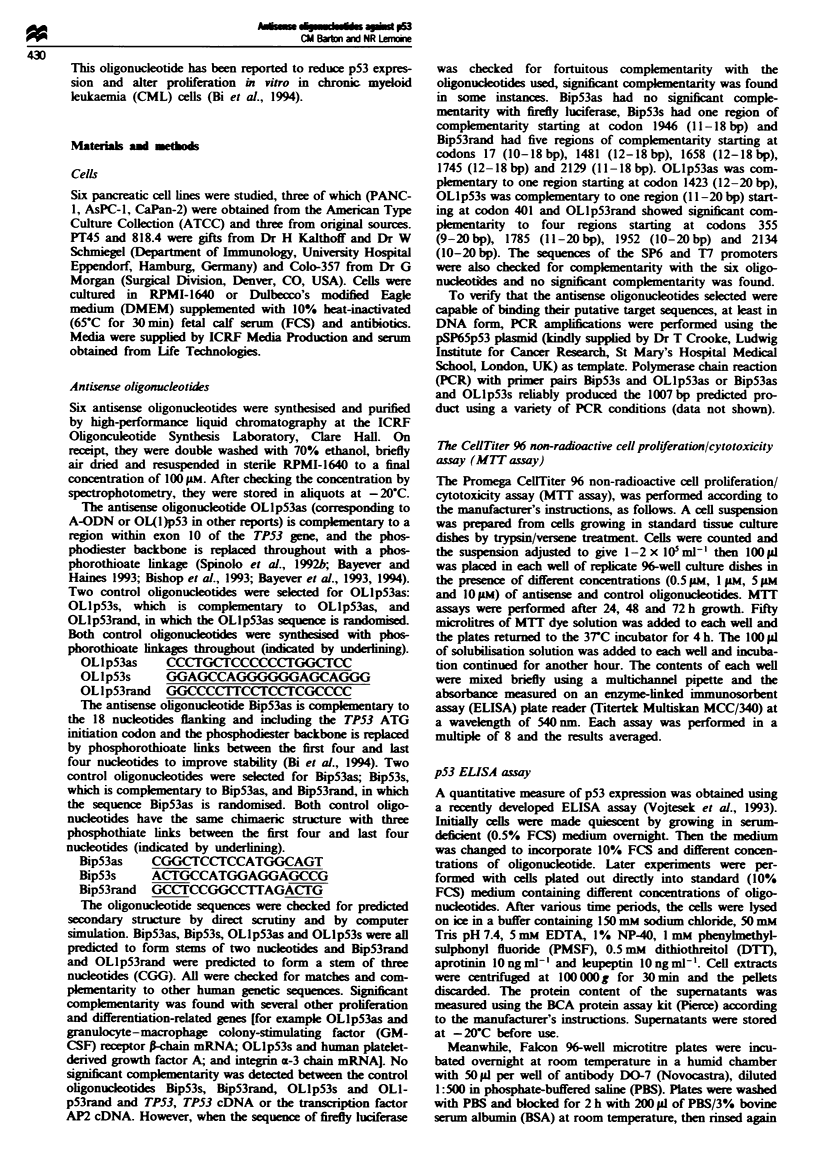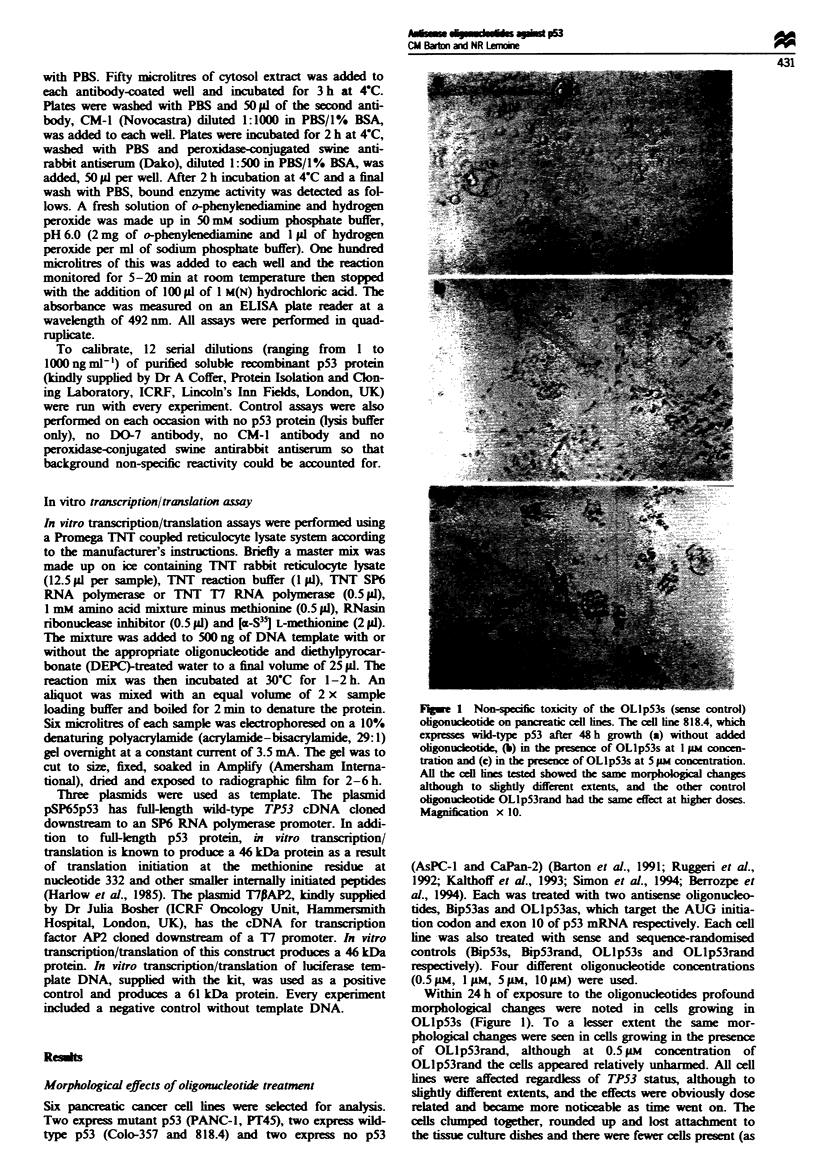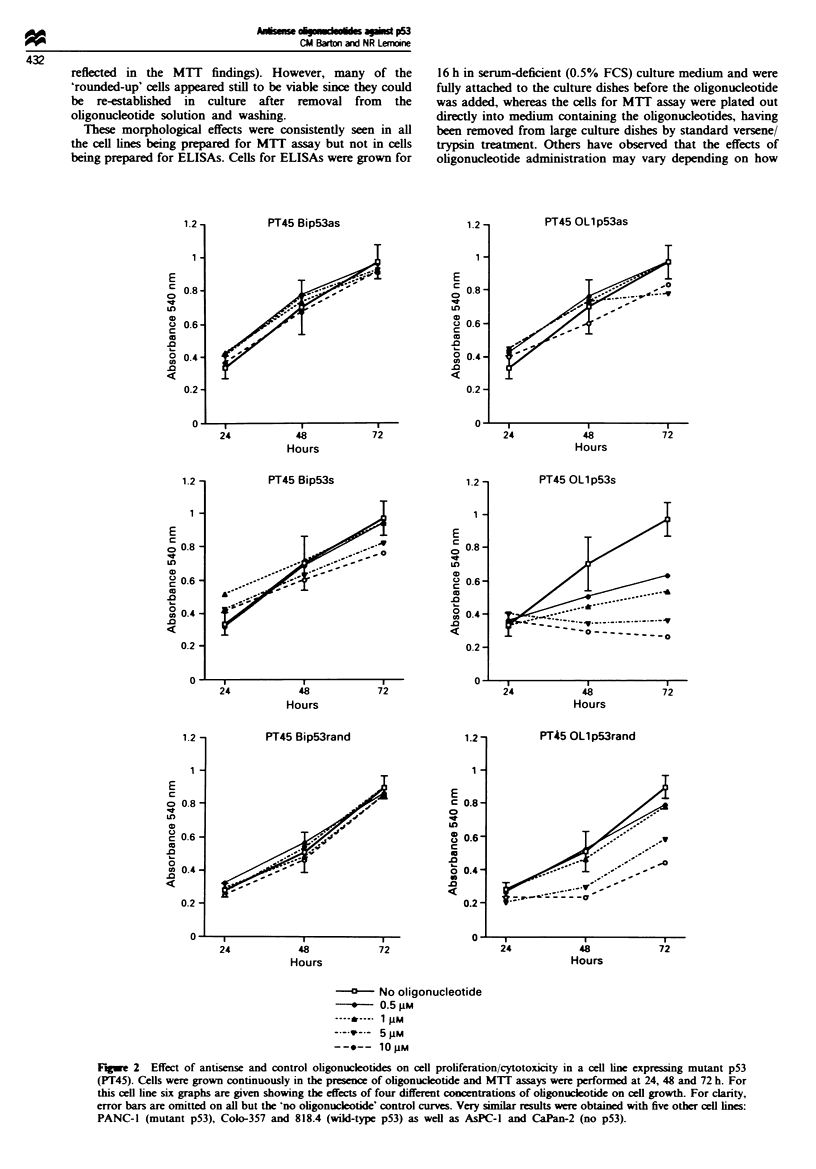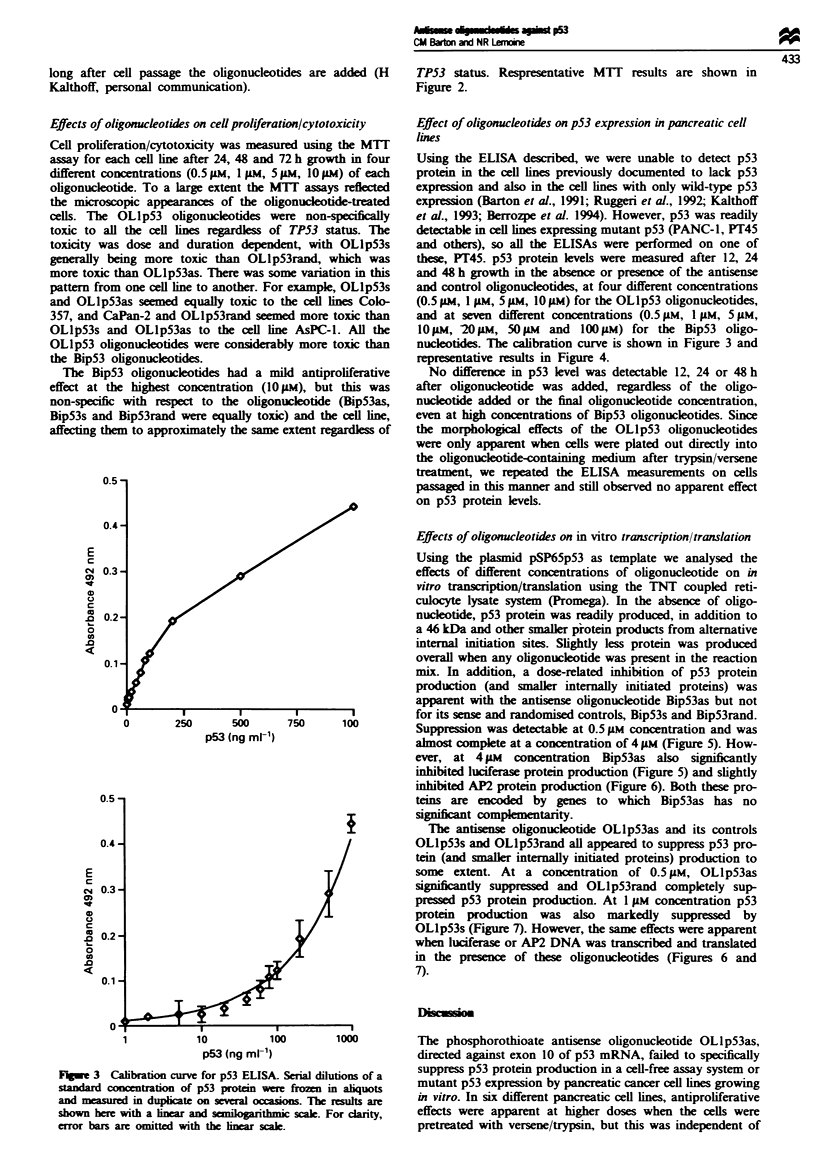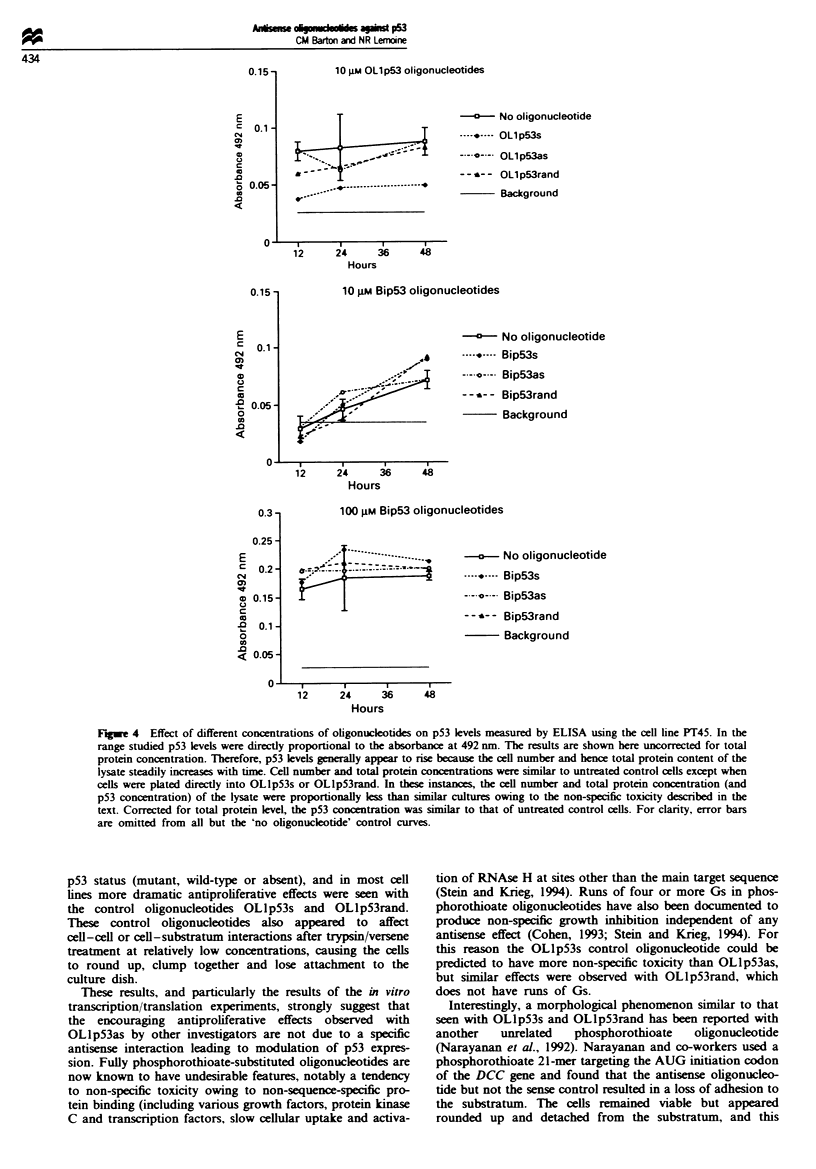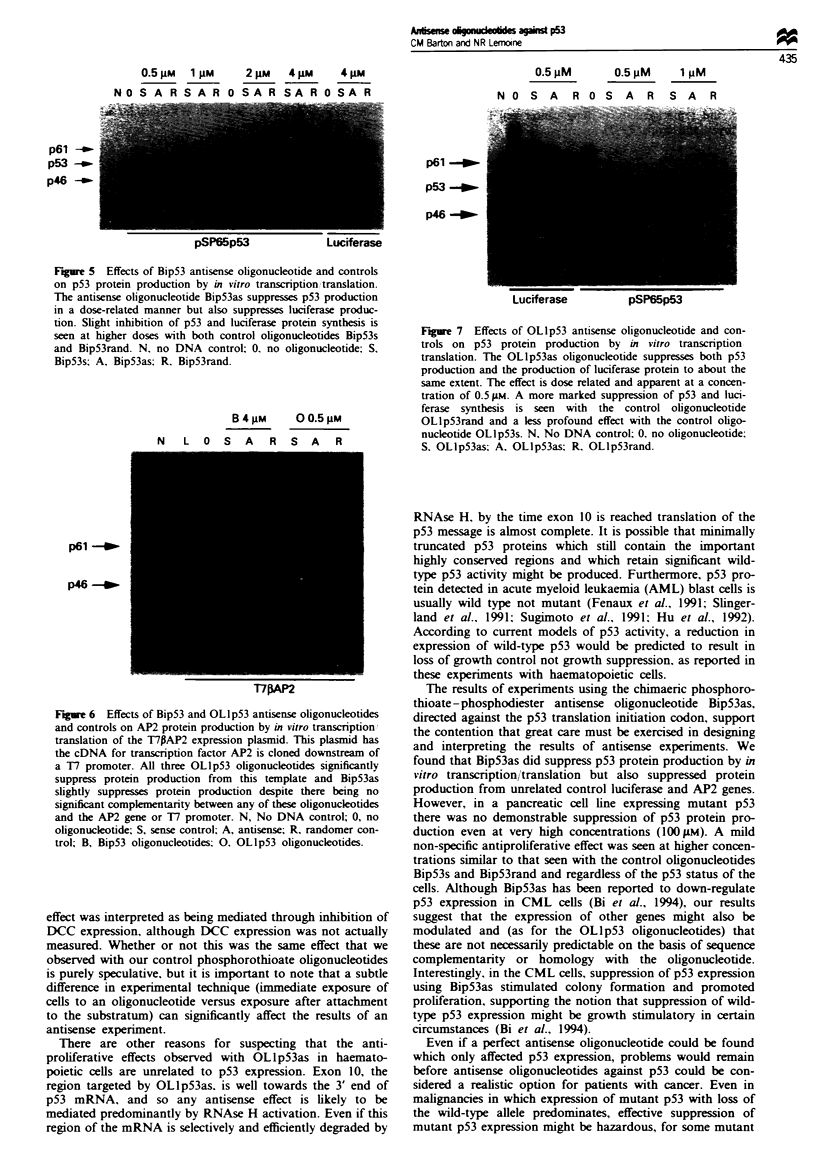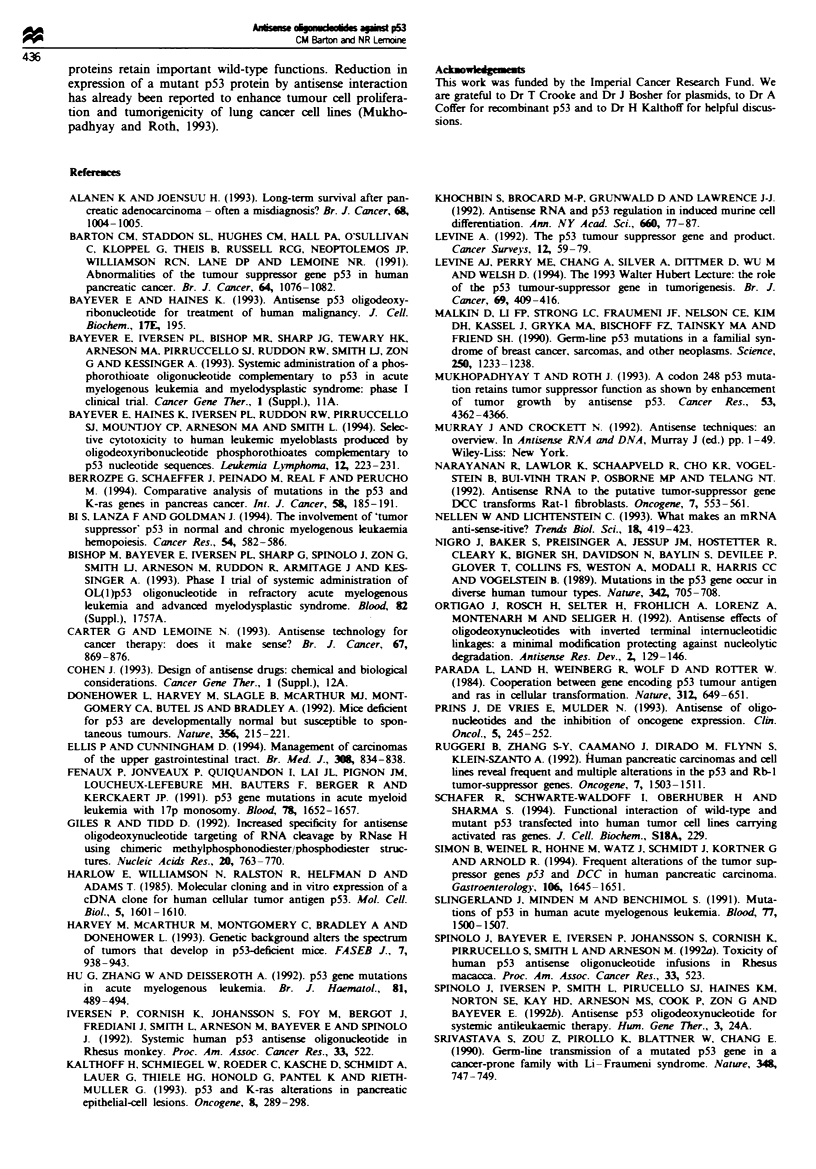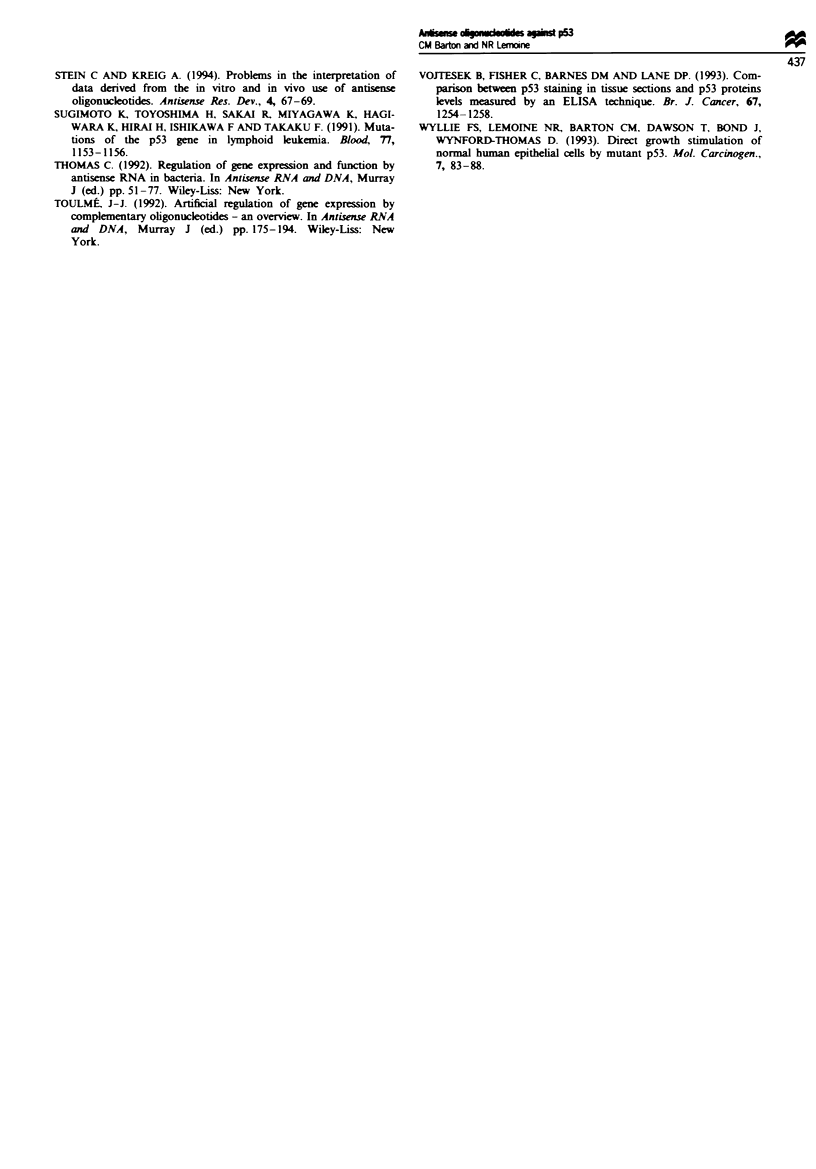# Antisense oligonucleotides directed against p53 have antiproliferative effects unrelated to effects on p53 expression.

**DOI:** 10.1038/bjc.1995.88

**Published:** 1995-03

**Authors:** C. M. Barton, N. R. Lemoine

**Affiliations:** Imperial Cancer Research Fund Oncology Unit, Hammersmith Hospital, London, UK.

## Abstract

**Images:**


British Journal of Cancer (1995) 71, 429-437

© 1995 Stockton Press All rights reserved 0007-0920/95 $9.00

### Antisense oligonucleotides directed against p53 have antiproliferative effects unrelated to effects on p53 expression

CM Barton and NR Lemoine

*Molecular Pathology Laboratory, Imperial Cancer Research Fund Oncology Unit, Hammersmith Hospital, Du Cane Road, London W12 0NN, UK.*

**Summary** Antisense oligonucleotides targeting p53 have been hailed as a potentially new technique for treating patients with cancer, and there have been encouraging reports of good patient tolerance *in vivo* and of antiproliferative effects *in vitro*. However, evidence is lacking that these oligonucleotides are acting via an antisense interaction to modulate p53 expression. We examined a phosphorothioate antisense oligonucleotide, directed against exon 10 of the *TP53* gene, and a chimaeric phosphorothioate-phosphodiester oligonucleotide directed against the p53 translation initiation codon. Both failed to specifically suppress p53 protein production in a cell-free assay system or to have any effect on mutant p53 expression by human pancreatic cancer cell lines. Antiproliferative effects were apparent, especially with the phosphorothioate antisense oligonucleotide, but this was independent of the p53 status of the cells (mutant, wild-type or absent) and also occurred with the control (sense and randomised) oligonucleotides. The most dramatic antiproliferative effects were seen with the 'control' phosphorothioate oligonucleotides. These findings suggest that the antiproliferative effects of some antisense oligonucleotides may be unrelated to expression of the gene they have been designed to target.

**Keywords:** antisense; oligonucleotide; p53; cancer therapy

Modulation of gene expression by naturally occurring antisense interactions is well documented in prokaryotes and may occur naturally in eukaryotic cells (reviewed in Murray and Crockett, 1992; Thomas, 1992; Nellen and Lichtenstein, 1993). Antisense techniques have also been devised to selectively reduce gene expression by the sequence-specific binding of complementary nucleic acids. Such techniques have become powerful tools for selectively reducing the expression of target genes *in vitro*, and there is increasing interest in the possibility of using the same technology *in vivo* for therapeutic purposes.

The antisense oligonucleotide approach involves the exogenous administration of short, synthetic, single-stranded oligonucleotide sequences, generally DNA based, which are taken up relatively inefficiently by the cell and released into the cytoplasm. They are thought to act predominantly by blocking translation of mRNA (reviewed in Murray and Crockett, 1992; Toulmé, 1992; Carter and Lemoine, 1993; Nellen and Lichtenstein, 1993; Prins *et al.*, 1993). Sequences complementary to the protein-coding part of the mRNA are thought to act mainly by causing cleavage by RNAse H, which specifically degrades the RNA component of RNA-DNA hybrids. Sequences which target the 5′ untranslated region are thought to prevent attachment and sliding of the 40S ribosomal subunit by steric hindrance, and those which bind close to the AUG initiation codon may prevent further assembly of the translation initiation complex.

Most work has been done with oligonucleotides modified to increase their stability and lipid solubility, for example by replacement of the phosphodiester linkage in the sugar-phosphate backbone to increase nuclease resistance. A phosphorothiorate linkage (in which the oxygen atom is replaced by a sulphur atom) is a commonly used modification. Chimaeric oligonucleotides have the modified linkage for only part of the molecule, usually the 5′ and 3′ ends, since intracellular degradation is mainly due to exonucleases (Giles and Tidd, 1992; Ortigao *et al.*, 1992; Toulmé, 1992).

*TP53* is an attractive target for an antisense approach in human cancer for several reasons. Although classified as a tumour-suppressor gene, the mutant forms commonly found in human tumours have many of the features associated with a dominant oncogene, including transforming activity (Parada *et al.*, 1984). *TP53* is the most frequent gene to be mutated in human cancer, and expression of mutant p53 is thought to be important in a wide range of tumours, including many that are primarily resistant to conventional forms of therapy (Nigro *et al.*, 1989; Levine, 1992; Levine *et al.*, 1994). Wild-type p53 is dispensable for normal cell growth and metabolism, albeit at the cost of an increased susceptibility to malignant change (Malkin *et al.*, 1990; Srivastava *et al.*, 1990; Donehower *et al.*, 1992; Harvey *et al.*, 1993). There is also evidence for a gene dosage effect so that complete suppression of mutant p53 expression may not be necessary for a significant anti-tumour effect, especially if there is some residual wild-type gene expression (Schafer *et al.*, 1994). Lastly, there is evidence that p53 expression may be modulated naturally by the endogenous production of antisense RNA transcripts (Khochbin *et al.*, 1992).

Phosphorothiate oligonucleotides have now been given systemically to animals and humans (Iversen *et al.*, 1992; Spinolo *et al.*, 1992*a*). In a phase I clinical trial a phosphorothioate oligonucleotide targeting p53 exon 10, OL(1) p53, was given by continuous infusion for up to 10 days and was well tolerated with few adverse effects (Spinolo *et al.*, 1992*b*, Bishop *et al.*, 1993; Bayever *et al.*, 1993, 1994). This oligonucleotide has been reported to have a significant antiproliferative effect on AML (Bayever *et al.*, 1994) and pancreatic cancer cells (Bayever and Haines, 1993) *in vitro*, and, when given by systemic infusion to patients, to inhibit the growth of their leukaemic blasts *in vitro* (Spinolo *et al.*, 1992*b*; Bishop *et al.*, 1993; Bayever *et al.*, 1993, 1994). Exciting as these reports are, no evidence has been provided that the oligonucleotide is acting via an antisense interaction or that it modulates p53 expression. We have investigated this oligonucleotide in pancreatic cancer cell lines, since expression of mutant p53 is thought to be an important early event in pancreatic carcinogenesis (Barton *et al.*, 1991; Wyllie *et al.*, 1993) and because pancreatic cancer is notoriously resistant to currently available cancer therapies (Alanen and Joensuu, 1993; Ellis and Cunningham, 1994). For comparison, we have also investigated a chimaeric phosphorothioate-phosphodiester antisense oligonucleotide which targets a different region of p53 mRNA, the initiation codon.

Correspondence: NR Lemoine
Received 29 July 1994; revised 7 October 1994; accepted 14 October 1994

This oligonucleotide has been reported to reduce p53 expression and alter proliferation *in vitro* in chronic myeloid leukaemia (CML) cells (Bi *et al.*, 1994).

### Materials and methods

### *Cells*

Six pancreatic cell lines were studied, three of which (PANC-1, AsPC-1, CaPan-2) were obtained from the American Type Culture Collection (ATCC) and three from original sources. PT45 and 818.4 were gifts from Dr H Kalthoff and Dr W Schmiegel (Department of Immunology, University Hospital Eppendorf, Hamburg, Germany) and Colo-357 from Dr G Morgan (Surgical Division, Denver, CO, USA). Cells were cultured in RPMI-1640 or Dulbecco's modified Eagle medium (DMEM) supplemented with 10% heat-inactivated (65°C for 30 min) fetal calf serum (FCS) and antibiotics. Media were supplied by ICRF Media Production and serum obtained from Life Technologies.

### *Antisense oligonucleotides*

Six antisense oligonucleotides were synthesised and purified by high-performance liquid chromatography at the ICRF Oligonculeotide Synthesis Laboratory, Clare Hall. On receipt, they were double washed with 70% ethanol, briefly air dried and resuspended in sterile RPMI-1640 to a final concentration of 100 μM. After checking the concentration by spectrophotometry, they were stored in aliquots at −20°C.

The antisense oligonucleotide OL1p53as (corresponding to A-ODN or OL(1)p53 in other reports) is complementary to a region within exon 10 of the *TP53* gene, and the phosphodiester backbone is replaced throughout with a phosphorothioate linkage (Spinolo *et al.*, 1992*b*; Bayever and Haines 1993; Bishop *et al.*, 1993; Bayever *et al.*, 1993, 1994). Two control oligonucleotides were selected for OL1p53as: OL1p53s, which is complementary to OL1p53as, and OL1p53rand, in which the OL1p53as sequence is randomised. Both control oligonucleotides were synthesised with phosphorothioate linkages throughout (indicated by underlining).

| | |
|---|---|
| OL1p53as | CCCTGCTCCCCCCTGGCTCC |
| OL1p53s | GGAGCCAGGGGGGAGCAGGG |
| OL1p53rand | GGCCCCTTCCTCCTCGCCCC |

The antisense oligonucleotide Bip53as is complementary to the 18 nucleotides flanking and including the *TP53* ATG initiation codon and the phosphodiester backbone is replaced by phosphorothioate links between the first four and last four nucleotides to improve stability (Bi *et al.*, 1994). Two control oligonucleotides were selected for Bip53as; Bip53s, which is complementary to Bip53as, and Bip53rand, in which the sequence Bip53as is randomised. Both control oligonucleotides have the same chimaeric structure with three phosphothiate links between the first four and last four nucleotides (indicated by underlining).

| | |
|---|---|
| Bip53as | CGGCTCCTCCATGGCAGT |
| Bip53s | ACTGCCATGGAGGAGCCG |
| Bip53rand | GCCTCCGGCCTTAGACTG |

The oligonucleotide sequences were checked for predicted secondary structure by direct scrutiny and by computer simulation. Bip53as, Bip53s, OL1p53as and OL1p53s were all predicted to form stems of two nucleotides and Bip53rand and OL1p53rand were predicted to form a stem of three nucleotides (CGG). All were checked for matches and complementarity to other human genetic sequences. Significant complementarity was found with several other proliferation and differentiation-related genes [for example OL1p53as and granulocyte–macrophage colony-stimulating factor (GM-CSF) receptor β-chain mRNA; OL1p53s and human platelet-derived growth factor A; and integrin α-3 chain mRNA]. No significant complementarity was detected between the control oligonucleotides Bip53s, Bip53rand, OL1p53s and OL1-p53rand and *TP53*, *TP53* cDNA or the transcription factor AP2 cDNA. However, when the sequence of firefly luciferase was checked for fortuitous complementarity with the oligonucleotides used, significant complementarity was found in some instances. Bip53as had no significant complementarity with firefly luciferase, Bip53s had one region of complementarity starting at codon 1946 (11–18 bp) and Bip53rand had five regions of complementarity starting at codons 17 (10–18 bp), 1481 (12–18 bp), 1658 (12–18 bp), 1745 (12–18 bp) and 2129 (11–18 bp). OL1p53as was complementary to one region starting at codon 1423 (12–20 bp), OL1p53s was complementary to one region (11–20 bp) starting at codon 401 and OL1p53rand showed significant complementarity to four regions starting at codons 355 (9–20 bp), 1785 (11–20 bp), 1952 (10–20 bp) and 2134 (10–20 bp). The sequences of the SP6 and T7 promoters were also checked for complementarity with the six oligonucleotides and no significant complementarity was found.

To verify that the antisense oligonucleotides selected were capable of binding their putative target sequences, at least in DNA form, PCR amplifications were performed using the pSP65p53 plasmid (kindly supplied by Dr T Crooke, Ludwig Institute for Cancer Research, St Mary's Hospital Medical School, London, UK) as template. Polymerase chain reaction (PCR) with primer pairs Bip53s and OL1p53as or Bip53as and OL1p53s reliably produced the 1007 bp predicted product using a variety of PCR conditions (data not shown).

### *The CellTiter 96 non-radioactive cell proliferation/cytotoxicity assay (MTT assay)*

The Promega CellTiter 96 non-radioactive cell proliferation/cytotoxicity assay (MTT assay), was performed according to the manufacturer's instructions, as follows. A cell suspension was prepared from cells growing in standard tissue culture dishes by trypsin/versene treatment. Cells were counted and the suspension adjusted to give $1-2\times10^5$ ml$^{-1}$ then 100 μl was placed in each well of replicate 96-well culture dishes in the presence of different concentrations (0.5 μM, 1 μM, 5 μM and 10 μM) of antisense and control oligonucleotides. MTT assays were performed after 24, 48 and 72 h growth. Fifty microlitres of MTT dye solution was added to each well and the plates returned to the 37°C incubator for 4 h. The 100 μl of solubilisation solution was added to each well and incubation continued for another hour. The contents of each well were mixed briefly using a multichannel pipette and the absorbance measured on an enzyme-linked immunosorbent assay (ELISA) plate reader (Titertek Multiskan MCC/340) at a wavelength of 540 nm. Each assay was performed in a multiple of 8 and the results averaged.

### *p53 ELISA assay*

A quantitative measure of p53 expression was obtained using a recently developed ELISA assay (Vojtesek *et al.*, 1993). Initially cells were made quiescent by growing in serum-deficient (0.5% FCS) medium overnight. Then the medium was changed to incorporate 10% FCS and different concentrations of oligonucleotide. Later experiments were performed with cells plated out directly into standard (10% FCS) medium containing different concentrations of oligonucleotides. After various time periods, the cells were lysed on ice in a buffer containing 150 mM sodium chloride, 50 mM Tris pH 7.4, 5 mM EDTA, 1% NP-40, 1 mM phenylmethylsulphonyl fluoride (PMSF), 0.5 mM dithiothreitol (DTT), aprotinin 10 ng ml$^{-1}$ and leupeptin 10 ng ml$^{-1}$. Cell extracts were centrifuged at 100 000 *g* for 30 min and the pellets discarded. The protein content of the supernatants was measured using the BCA protein assay kit (Pierce) according to the manufacturer's instructions. Supernatants were stored at −20°C before use.

Meanwhile, Falcon 96-well microtitre plates were incubated overnight at room temperature in a humid chamber with 50 μl per well of antibody DO-7 (Novocastra), diluted 1:500 in phosphate-buffered saline (PBS). Plates were washed with PBS and blocked for 2 h with 200 μl of PBS/3% bovine serum albumin (BSA) at room temperature, then rinsed again with PBS. Fifty microlitres of cytosol extract was added to each antibody-coated well and incubated for 3 h at 4°C. Plates were washed with PBS and 50 µl of the second antibody, CM-1 (Novocastra) diluted 1:1000 in PBS/1% BSA, was added to each well. Plates were incubated for 2 h at 4°C, washed with PBS and peroxidase-conjugated swine antirabbit antiserum (Dako), diluted 1:500 in PBS/1% BSA, was added, 50 µl per well. After 2 h incubation at 4°C and a final wash with PBS, bound enzyme activity was detected as follows. A fresh solution of *o*-phenylenediamine and hydrogen peroxide was made up in 50 mM sodium phosphate buffer, pH 6.0 (2 mg of *o*-phenylenediamine and 1 µl of hydrogen peroxide per ml of sodium phosphate buffer). One hundred microlitres of this was added to each well and the reaction monitored for 5–20 min at room temperature then stopped with the addition of 100 µl of 1 M(N) hydrochloric acid. The absorbance was measured on an ELISA plate reader at a wavelength of 492 nm. All assays were performed in quadruplicate.

To calibrate, 12 serial dilutions (ranging from 1 to 1000 ng $ml^{-1}$) of purified soluble recombinant p53 protein (kindly supplied by Dr A Coffer, Protein Isolation and Cloning Laboratory, ICRF, Lincoln's Inn Fields, London, UK) were run with every experiment. Control assays were also performed on each occasion with no p53 protein (lysis buffer only), no DO-7 antibody, no CM-1 antibody and no peroxidase-conjugated swine antirabbit antiserum so that background non-specific reactivity could be accounted for.

### In vitro *transcription/translation assay*

*In vitro* transcription/translation assays were performed using a Promega TNT coupled reticulocyte lysate system according to the manufacturer's instructions. Briefly a master mix was made up on ice containing TNT rabbit reticulocyte lysate (12.5 µl per sample), TNT reaction buffer (1 µl), TNT SP6 RNA polymerase or TNT T7 RNA polymerase (0.5 µl), 1 mM amino acid mixture minus methionine (0.5 µl), RNasin ribonuclease inhibitor (0.5 µl) and [α-$S^{35}$] L-methionine (2 µl). The mixture was added to 500 ng of DNA template with or without the appropriate oligonucleotide and diethylpyrocarbonate (DEPC)-treated water to a final volume of 25 µl. The reaction mix was then incubated at 30°C for 1–2 h. An aliquot was mixed with an equal volume of 2 × sample loading buffer and boiled for 2 min to denature the protein. Six microlitres of each sample was electrophoresed on a 10% denaturing polyacrylamide (acrylamide–bisacrylamide, 29:1) gel overnight at a constant current of 3.5 mA. The gel was to cut to size, fixed, soaked in Amplify (Amersham International), dried and exposed to radiographic film for 2–6 h.

Three plasmids were used as template. The plasmid pSP65p53 has full-length wild-type *TP53* cDNA cloned downstream to an SP6 RNA polymerase promoter. In addition to full-length p53 protein, *in vitro* transcription/translation is known to produce a 46 kDa protein as a result of translation initiation at the methionine residue at nucleotide 332 and other smaller internally initiated peptides (Harlow *et al.*, 1985). The plasmid T7βAP2, kindly supplied by Dr Julia Bosher (ICRF Oncology Unit, Hammersmith Hospital, London, UK), has the cDNA for transcription factor AP2 cloned downstream of a T7 promoter. *In vitro* transcription/translation of this construct produces a 46 kDa protein. *In vitro* transcription/translation of luciferase template DNA, supplied with the kit, was used as a positive control and produces a 61 kDa protein. Every experiment included a negative control without template DNA.

### Results

### *Morphological effects of oligonucleotide treatment*

Six pancreatic cancer cell lines were selected for analysis. Two express mutant p53 (PANC-1, PT45), two express wild-type p53 (Colo-357 and 818.4) and two express no p53 (AsPC-1 and CaPan-2) (Barton *et al.*, 1991; Ruggeri *et al.*, 1992; Kalthoff *et al.*, 1993; Simon *et al.*, 1994; Berrozpe *et al.*, 1994). Each was treated with two antisense oligonucleotides, Bip53as and OL1p53as, which target the AUG initiation codon and exon 10 of p53 mRNA respectively. Each cell line was also treated with sense and sequence-randomised controls (Bip53s, Bip53rand, OL1p53s and OL1p53rand respectively). Four different oligonucleotide concentrations (0.5 µM, 1 µM, 5 µM, 10 µM) were used.

Within 24 h of exposure to the oligonucleotides profound morphological changes were noted in cells growing in OL1p53s (Figure 1). To a lesser extent the same morphological changes were seen in cells growing in the presence of OL1p53rand, although at 0.5 µM concentration of OL1p53rand the cells appeared relatively unharmed. All cell lines were affected regardless of *TP53* status, although to slightly different extents, and the effects were obviously dose related and became more noticeable as time went on. The cells clumped together, rounded up and lost attachment to the tissue culture dishes and there were fewer cells present (as

**Figure 1** Non-specific toxicity of the OL1p53s (sense control) oligonucleotide on pancreatic cell lines. The cell line 818.4, which expresses wild-type p53 after 48 h growth (**a**) without added oligonucleotide, (**b**) in the presence of OL1p53s at 1 µM concentration and (**c**) in the presence of OL1p53s at 5 µM concentration. All the cell lines tested showed the same morphological changes although to slightly different extents, and the other control oligonucleotide OL1p53rand had the same effect at higher doses. Magnification × 10.

reflected in the MTT findings). However, many of the 'rounded-up' cells appeared still to be viable since they could be re-established in culture after removal from the oligonucleotide solution and washing.

These morphological effects were consistently seen in all the cell lines being prepared for MTT assay but not in cells being prepared for ELISAs. Cells for ELISAs were grown for 16 h in serum-deficient (0.5% FCS) culture medium and were fully attached to the culture dishes before the oligonucleotide was added, whereas the cells for MTT assay were plated out directly into medium containing the oligonucleotides, having been removed from large culture dishes by standard versene/trypsin treatment. Others have observed that the effects of oligonucleotide administration may vary depending on how

**Figure 2** Effect of antisense and control oligonucleotides on cell proliferation/cytotoxicity in a cell line expressing mutant p53 (PT45). Cells were grown continuously in the presence of oligonucleotide and MTT assays were performed at 24, 48 and 72 h. For this cell line six graphs are given showing the effects of four different concentrations of oligonucleotide on cell growth. For clarity, error bars are omitted on all but the 'no oligonucleotide' control curves. Very similar results were obtained with five other cell lines: PANC-1 (mutant p53), Colo-357 and 818.4 (wild-type p53) as well as AsPC-1 and CaPan-2 (no p53).

long after cell passage the oligonucleotides are added (H Kalthoff, personal communication).

*Effects of oligonucleotides on cell proliferation/cytotoxicity*

Cell proliferation/cytotoxicity was measured using the MTT assay for each cell line after 24, 48 and 72 h growth in four different concentrations (0.5 μM, 1 μM, 5 μM, 10 μM) of each oligonucleotide. To a large extent the MTT assays reflected the microscopic appearances of the oligonucleotide-treated cells. The OL1p53 oligonucleotides were non-specifically toxic to all the cell lines regardless of *TP53* status. The toxicity was dose and duration dependent, with OL1p53s generally being more toxic than OL1p53rand, which was more toxic than OL1p53as. There was some variation in this pattern from one cell line to another. For example, OL1p53s and OL1p53as seemed equally toxic to the cell lines Colo-357, and CaPan-2 and OL1p53rand seemed more toxic than OL1p53s and OL1p53as to the cell line AsPC-1. All the OL1p53 oligonucleotides were considerably more toxic than the Bip53 oligonucleotides.

The Bip53 oligonucleotides had a mild antiproliferative effect at the highest concentration (10 μM), but this was non-specific with respect to the oligonucleotide (Bip53as, Bip53s and Bip53rand were equally toxic) and the cell line, affecting them to approximately the same extent regardless of

**Figure 3** Calibration curve for p53 ELISA. Serial dilutions of a standard concentration of p53 protein were frozen in aliquots and measured in duplicate on several occasions. The results are shown here with a linear and semilogarithmic scale. For clarity, error bars are omitted with the linear scale.

*TP53* status. Respresentative MTT results are shown in Figure 2.

*Effect of oligonucleotides on p53 expression in pancreatic cell lines*

Using the ELISA described, we were unable to detect p53 protein in the cell lines previously documented to lack p53 expression and also in the cell lines with only wild-type p53 expression (Barton *et al.*, 1991; Ruggeri *et al.*, 1992; Kalthoff *et al.*, 1993; Berrozpe *et al.* 1994). However, p53 was readily detectable in cell lines expressing mutant p53 (PANC-1, PT45 and others), so all the ELISAs were performed on one of these, PT45. p53 protein levels were measured after 12, 24 and 48 h growth in the absence or presence of the antisense and control oligonucleotides, at four different concentrations (0.5 μM, 1 μM, 5 μM, 10 μM) for the OL1p53 oligonucleotides, and at seven different concentrations (0.5 μM, 1 μM, 5 μM, 10 μM, 20 μM, 50 μM and 100 μM) for the Bip53 oligonucleotides. The calibration curve is shown in Figure 3 and representative results in Figure 4.

No difference in p53 level was detectable 12, 24 or 48 h after oligonucleotide was added, regardless of the oligonucleotide added or the final oligonucleotide concentration, even at high concentrations of Bip53 oligonucleotides. Since the morphological effects of the OL1p53 oligonucleotides were only apparent when cells were plated out directly into the oligonucleotide-containing medium after trypsin/versene treatment, we repeated the ELISA measurements on cells passaged in this manner and still observed no apparent effect on p53 protein levels.

*Effects of oligonucleotides on* in vitro *transcription/translation*

Using the plasmid pSP65p53 as template we analysed the effects of different concentrations of oligonucleotide on *in vitro* transcription/translation using the TNT coupled reticulocyte lysate system (Promega). In the absence of oligonucleotide, p53 protein was readily produced, in addition to a 46 kDa and other smaller protein products from alternative internal initiation sites. Slightly less protein was produced overall when any oligonucleotide was present in the reaction mix. In addition, a dose-related inhibition of p53 protein production (and smaller internally initiated proteins) was apparent with the antisense oligonucleotide Bip53as but not for its sense and randomised controls, Bip53s and Bip53rand. Suppression was detectable at 0.5 μM concentration and was almost complete at a concentration of 4 μM (Figure 5). However, at 4 μM concentration Bip53as also significantly inhibited luciferase protein production (Figure 5) and slightly inhibited AP2 protein production (Figure 6). Both these proteins are encoded by genes to which Bip53as has no significant complementarity.

The antisense oligonucleotide OL1p53as and its controls OL1p53s and OL1p53rand all appeared to suppress p53 protein (and smaller internally initiated proteins) production to some extent. At a concentration of 0.5 μM, OL1p53as significantly suppressed and OL1p53rand completely suppressed p53 protein production. At 1 μM concentration p53 protein production was also markedly suppressed by OL1p53s (Figure 7). However, the same effects were apparent when luciferase or AP2 DNA was transcribed and translated in the presence of these oligonucleotides (Figures 6 and 7).

### Discussion

The phosphorothioate antisense oligonucleotide OL1p53as, directed against exon 10 of p53 mRNA, failed to specifically suppress p53 protein production in a cell-free assay system or mutant p53 expression by pancreatic cancer cell lines growing *in vitro*. In six different pancreatic cell lines, antiproliferative effects were apparent at higher doses when the cells were pretreated with versene/trypsin, but this was independent of

**Figure 4** Effect of different concentrations of oligonucleotides on p53 levels measured by ELISA using the cell line PT45. In the range studied p53 levels were directly proportional to the absorbance at 492 nm. The results are shown here uncorrected for total protein concentration. Therefore, p53 levels generally appear to rise because the cell number and hence total protein content of the lysate steadily increases with time. Cell number and total protein concentrations were similar to untreated control cells except when cells were plated directly into OL1p53s or OL1p53rand. In these instances, the cell number and total protein concentration (and p53 concentration) of the lysate were proportionally less than similar cultures owing to the non-specific toxicity described in the text. Corrected for total protein level, the p53 concentration was similar to that of untreated control cells. For clarity, error bars are omitted from all but the 'no oligonucleotide' control curves.

p53 status (mutant, wild-type or absent), and in most cell lines more dramatic antiproliferative effects were seen with the control oligonucleotides OL1p53s and OL1p53rand. These control oligonucleotides also appeared to affect cell–cell or cell–substratum interactions after trypsin/versene treatment at relatively low concentrations, causing the cells to round up, clump together and lose attachment to the culture dish.

These results, and particularly the results of the *in vitro* transcription/translation experiments, strongly suggest that the encouraging antiproliferative effects observed with OL1p53as by other investigators are not due to a specific antisense interaction leading to modulation of p53 expression. Fully phosphorothioate-substituted oligonucleotides are now known to have undesirable features, notably a tendency to non-specific toxicity owing to non-sequence-specific protein binding (including various growth factors, protein kinase C and transcription factors, slow cellular uptake and activation of RNAse H at sites other than the main target sequence (Stein and Krieg, 1994). Runs of four or more Gs in phosphorothioate oligonucleotides have also been documented to produce non-specific growth inhibition independent of any antisense effect (Cohen, 1993; Stein and Krieg, 1994). For this reason the OL1p53s control oligonucleotide could be predicted to have more non-specific toxicity than OL1p53as, but similar effects were observed with OL1p53rand, which does not have runs of Gs.

Interestingly, a morphological phenomenon similar to that seen with OL1p53s and OL1p53rand has been reported with another unrelated phosphorothioate oligonucleotide (Narayanan *et al.*, 1992). Narayanan and co-workers used a phosphorothioate 21-mer targeting the AUG initiation codon of the *DCC* gene and found that the antisense oligonucleotide but not the sense control resulted in a loss of adhesion to the substratum. The cells remained viable but appeared rounded up and detached from the substratum, and this

**Figure 5** Effects of Bip53 antisense oligonucleotide and controls on p53 protein production by *in vitro* transcription/translation. The antisense oligonucleotide Bip53as suppresses p53 production in a dose-related manner but also suppresses luciferase production. Slight inhibition of p53 and luciferase protein synthesis is seen at higher doses with both control oligonucleotides Bip53s and Bip53rand. N, no DNA control; 0, no oligonucleotide; S, Bip53s; A, Bip53as; R, Bip53rand.

**Figure 7** Effects of OL1p53 antisense oligonucleotide and controls on p53 protein production by *in vitro* transcription/translation. The OL1p53as oligonucleotide suppresses both p53 production and the production of luciferase protein to about the same extent. The effect is dose related and apparent at a concentration of 0.5 µM. A more marked suppression of p53 and luciferase synthesis is seen with the control oligonucleotide OL1p53rand and a less profound effect with the control oligonucleotide OL1p53s. N, No DNA control; 0, no oligonucleotide; S, OL1p53as; A, OL1p53as; R, OL1p53rand.

**Figure 6** Effects of Bip53 and OL1p53 antisense oligonucleotides and controls on AP2 protein production by *in vitro* transcription/translation of the T7βAP2 expression plasmid. This plasmid has the cDNA for transcription factor AP2 is cloned downstream of a T7 promoter. All three OL1p53 oligonucleotides significantly suppress protein production from this template and Bip53as slightly suppresses protein production despite there being no significant complementarity between any of these oligonucleotides and the AP2 gene or T7 promoter. N, No DNA control; 0, no oligonucleotide; S, sense control; A, antisense; R, randomer control; B, Bip53 oligonucleotides; O, OL1p53 oligonucleotides.

effect was interpreted as being mediated through inhibition of DCC expression, although DCC expression was not actually measured. Whether or not this was the same effect that we observed with our control phosphorothioate oligonucleotides is purely speculative, but it is important to note that a subtle difference in experimental technique (immediate exposure of cells to an oligonucleotide versus exposure after attachment to the substratum) can significantly affect the results of an antisense experiment.

There are other reasons for suspecting that the antiproliferative effects observed with OL1p53as in haematopoietic cells are unrelated to p53 expression. Exon 10, the region targeted by OL1p53as, is well towards the 3′ end of p53 mRNA, and so any antisense effect is likely to be mediated predominantly by RNAse H activation. Even if this region of the mRNA is selectively and efficiently degraded by RNAse H, by the time exon 10 is reached translation of the p53 message is almost complete. It is possible that minimally truncated p53 proteins which still contain the important highly conserved regions and which retain significant wild-type p53 activity might be produced. Furthermore, p53 protein detected in acute myeloid leukaemia (AML) blast cells is usually wild type not mutant (Fenaux *et al.*, 1991; Slingerland *et al.*, 1991; Sugimoto *et al.*, 1991; Hu *et al.*, 1992). According to current models of p53 activity, a reduction in expression of wild-type p53 would be predicted to result in loss of growth control not growth suppression, as reported in these experiments with haematopoietic cells.

The results of experiments using the chimaeric phosphorothioate–phosphodiester antisense oligonucleotide Bip53as, directed against the p53 translation initiation codon, support the contention that great care must be exercised in designing and interpreting the results of antisense experiments. We found that Bip53as did suppress p53 protein production by *in vitro* transcription/translation but also suppressed protein production from unrelated control luciferase and AP2 genes. However, in a pancreatic cell line expressing mutant p53 there was no demonstrable suppression of p53 protein production even at very high concentrations (100 µM). A mild non-specific antiproliferative effect was seen at higher concentrations similar to that seen with the control oligonucleotides Bip53s and Bip53rand and regardless of the p53 status of the cells. Although Bip53as has been reported to down-regulate p53 expression in CML cells (Bi *et al.*, 1994), our results suggest that the expression of other genes might also be modulated and (as for the OL1p53 oligonucleotides) that these are not necessarily predictable on the basis of sequence complementarity or homology with the oligonucleotide. Interestingly, in the CML cells, suppression of p53 expression using Bip53as stimulated colony formation and promoted proliferation, supporting the notion that suppression of wild-type p53 expression might be growth stimulatory in certain circumstances (Bi *et al.*, 1994).

Even if a perfect antisense oligonucleotide could be found which only affected p53 expression, problems would remain before antisense oligonucleotides against p53 could be considered a realistic option for patients with cancer. Even in malignancies in which expression of mutant p53 with loss of the wild-type allele predominates, effective suppression of mutant p53 expression might be hazardous, for some mutant proteins retain important wild-type functions. Reduction in expression of a mutant p53 protein by antisense interaction has already been reported to enhance tumour cell proliferation and tumorigenicity of lung cancer cell lines (Mukhopadhyay and Roth, 1993).

**Acknowledgements**
This work was funded by the Imperial Cancer Research Fund. We are grateful to Dr T Crooke and Dr J Bosher for plasmids, to Dr A Coffer for recombinant p53 and to Dr H Kalthoff for helpful discussions.

**References**

ALANEN K AND JOENSUU H. (1993). Long-term survival after pancreatic adenocarcinoma – often a misdiagnosis? *Br. J. Cancer*, **68**, 1004–1005.

BARTON CM, STADDON SL, HUGHES CM, HALL PA, O'SULLIVAN C, KLOPPEL G, THEIS B, RUSSELL RCG, NEOPTOLEMOS JP, WILLIAMSON RCN, LANE DP AND LEMOINE NR. (1991). Abnormalities of the tumour suppressor gene p53 in human pancreatic cancer. *Br. J. Cancer*, **64**, 1076–1082.

BAYEVER E AND HAINES K. (1993). Antisense p53 oligodeoxyribonucleotide for treatment of human malignancy. *J. Cell. Biochem.*, **17E**, 195.

BAYEVER E, IVERSEN PL, BISHOP MR, SHARP JG, TEWARY HK, ARNESON MA, PIRRUCCELLO SJ, RUDDON RW, SMITH LJ, ZON G AND KESSINGER A. (1993). Systemic administration of a phosphorothioate oligonucleotide complementary to p53 in acute myelogenous leukemia and myelodysplastic syndrome: phase I clinical trial. *Cancer Gene Ther.*, **1** (Suppl.), 11A.

BAYEVER E, HAINES K, IVERSEN PL, RUDDON RW, PIRRUCCELLO SJ, MOUNTJOY CP, ARNESON MA AND SMITH L. (1994). Selective cytotoxicity to human leukemic myeloblasts produced by oligodeoxyribonucleotide phosphorothioates complementary to p53 nucleotide sequences. *Leukemia Lymphoma*, **12**, 223–231.

BERROZPE G, SCHAEFFER J, PEINADO M, REAL F AND PERUCHO M. (1994). Comparative analysis of mutations in the p53 and K-ras genes in pancreas cancer. *Int. J. Cancer*, **58**, 185–191.

BI S, LANZA F AND GOLDMAN J. (1994). The involvement of 'tumor suppressor' p53 in normal and chronic myelogenous leukaemia hemopoiesis. *Cancer Res.*, **54**, 582–586.

BISHOP M, BAYEVER E, IVERSEN PL, SHARP G, SPINOLO J, ZON G, SMITH LJ, ARNESON M, RUDDON R, ARMITAGE J AND KESSINGER A. (1993). Phase I trial of systemic administration of OL(1)p53 oligonucleotide in refractory acute myelogenous leukemia and advanced myelodysplastic syndrome. *Blood*, **82** (Suppl.), 1757A.

CARTER G AND LEMOINE N. (1993). Antisense technology for cancer therapy: does it make sense? *Br. J. Cancer*, **67**, 869–876.

COHEN J. (1993). Design of antisense drugs: chemical and biological considerations. *Cancer Gene Ther.*, **1** (Suppl.), 12A.

DONEHOWER L, HARVEY M, SLAGLE B, MCARTHUR MJ, MONTGOMERY CA, BUTEL JS AND BRADLEY A. (1992). Mice deficient for p53 are developmentally normal but susceptible to spontaneous tumours. *Nature*, **356**, 215–221.

ELLIS P AND CUNNINGHAM D. (1994). Management of carcinomas of the upper gastrointestinal tract. *Br. Med. J.*, **308**, 834–838.

FENAUX P, JONVEAUX P, QUIQUANDON I, LAI JL, PIGNON JM, LOUCHEUX-LEFEBURE MH, BAUTERS F, BERGER R AND KERCKAERT JP. (1991). p53 gene mutations in acute myeloid leukemia with 17p monosomy. *Blood*, **78**, 1652–1657.

GILES R AND TIDD D. (1992). Increased specificity for antisense oligodeoxynucleotide targeting of RNA cleavage by RNase H using chimeric methylphosphonodiester/phosphodiester structures. *Nucleic Acids Res.*, **20**, 763–770.

HARLOW E, WILLIAMSON N, RALSTON R, HELFMAN D AND ADAMS T. (1985). Molecular cloning and in vitro expression of a cDNA clone for human cellular tumor antigen p53. *Mol. Cell. Biol.*, **5**, 1601–1610.

HARVEY M, MCARTHUR M, MONTGOMERY C, BRADLEY A AND DONEHOWER L. (1993). Genetic background alters the spectrum of tumors that develop in p53-deficient mice. *FASEB J.*, **7**, 938–943.

HU G, ZHANG W AND DEISSEROTH A. (1992). p53 gene mutations in acute myelogenous leukemia. *Br. J. Haematol.*, **81**, 489–494.

IVERSEN P, CORNISH K, JOHANSSON S, FOY M, BERGOT J, FREDIANI J, SMITH L, ARNESON M, BAYEVER E AND SPINOLO J. (1992). Systemic human p53 antisense oligonucleotide in Rhesus monkey. *Proc. Am. Assoc. Cancer Res.*, **33**, 522.

KALTHOFF H, SCHMIEGEL W, ROEDER C, KASCHE D, SCHMIDT A, LAUER G, THIELE HG, HONOLD G, PANTEL K AND RIETHMULLER G. (1993). p53 and K-ras alterations in pancreatic epithelial-cell lesions. *Oncogene*, **8**, 289–298.

KHOCHBIN S, BROCARD M-P, GRUNWALD D AND LAWRENCE J-J. (1992). Antisense RNA and p53 regulation in induced murine cell differentiation. *Ann. NY Acad. Sci.*, **660**, 77–87.

LEVINE A. (1992). The p53 tumour suppressor gene and product. *Cancer Surveys*, **12**, 59–79.

LEVINE AJ, PERRY ME, CHANG A, SILVER A, DITTMER D, WU M AND WELSH D. (1994). The 1993 Walter Hubert Lecture: the role of the p53 tumour-suppressor gene in tumorigenesis. *Br. J. Cancer*, **69**, 409–416.

MALKIN D, LI FP, STRONG LC, FRAUMENI JF, NELSON CE, KIM DH, KASSEL J, GRYKA MA, BISCHOFF FZ, TAINSKY MA AND FRIEND SH. (1990). Germ-line p53 mutations in a familial syndrome of breast cancer, sarcomas, and other neoplasms. *Science*, **250**, 1233–1238.

MUKHOPADHYAY T AND ROTH J. (1993). A codon 248 p53 mutation retains tumor suppressor function as shown by enhancement of tumor growth by antisense p53. *Cancer Res.*, **53**, 4362–4366.

MURRAY J AND CROCKETT N. (1992). Antisense techniques: an overview. In *Antisense RNA and DNA*, Murray J (ed.) pp. 1–49. Wiley-Liss: New York.

NARAYANAN R, LAWLOR K, SCHAAPVELD R, CHO KR, VOGELSTEIN B, BUI-VINH TRAN P, OSBORNE MP AND TELANG NT. (1992). Antisense RNA to the putative tumor-suppressor gene DCC transforms Rat-1 fibroblasts. *Oncogene*, **7**, 553–561.

NELLEN W AND LICHTENSTEIN C. (1993). What makes an mRNA anti-sense-itive? *Trends Biol. Sci.*, **18**, 419–423.

NIGRO J, BAKER S, PREISINGER A, JESSUP JM, HOSTETTER R, CLEARY K, BIGNER SH, DAVIDSON N, BAYLIN S, DEVILEE P, GLOVER T, COLLINS FS, WESTON A, MODALI R, HARRIS CC AND VOGELSTEIN B. (1989). Mutations in the p53 gene occur in diverse human tumour types. *Nature*, **342**, 705–708.

ORTIGAO J, ROSCH H, SELTER H, FROHLICH A, LORENZ A, MONTENARH M AND SELIGER H. (1992). Antisense effects of oligodeoxynucleotides with inverted terminal internucleotidic linkages: a minimal modification protecting against nucleolytic degradation. *Antisense Res. Dev.*, **2**, 129–146.

PARADA L, LAND H, WEINBERG R, WOLF D AND ROTTER W. (1984). Cooperation between gene encoding p53 tumour antigen and ras in cellular transformation. *Nature*, **312**, 649–651.

PRINS J, DE VRIES E, MULDER N. (1993). Antisense of oligonucleotides and the inhibition of oncogene expression. *Clin. Oncol.*, **5**, 245–252.

RUGGERI B, ZHANG S-Y, CAAMANO J, DIRADO M, FLYNN S, KLEIN-SZANTO A. (1992). Human pancreatic carcinomas and cell lines reveal frequent and multiple alterations in the p53 and Rb-1 tumor-suppressor genes. *Oncogene*, **7**, 1503–1511.

SCHAFER R, SCHWARTE-WALDOFF I, OBERHUBER H AND SHARMA S. (1994). Functional interaction of wild-type and mutant p53 transfected into human tumor cell lines carrying activated ras genes. *J. Cell. Biochem.*, **S18A**, 229.

SIMON B, WEINEL R, HOHNE M, WATZ J, SCHMIDT J, KORTNER G AND ARNOLD R. (1994). Frequent alterations of the tumor suppressor genes *p53* and *DCC* in human pancreatic carcinoma. *Gastroenterology*, **106**, 1645–1651.

SLINGERLAND J, MINDEN M AND BENCHIMOL S. (1991). Mutations of p53 in human acute myelogenous leukemia. *Blood*, **77**, 1500–1507.

SPINOLO J, BAYEVER E, IVERSEN P, JOHANSSON S, CORNISH K, PIRRUCELLO S, SMITH L AND ARNESON M. (1992*a*). Toxicity of human p53 antisense oligonucleotide infusions in Rhesus macacca. *Proc. Am. Assoc. Cancer Res.*, **33**, 523.

SPINOLO J, IVERSEN P, SMITH L, PIRUCELLO SJ, HAINES KM, NORTON SE, KAY HD, ARNESON MS, COOK P, ZON G AND BAYEVER E. (1992*b*). Antisense p53 oligodeoxynucleotide for systemic antileukaemic therapy. *Hum. Gene Ther.*, **3**, 24A.

SRIVASTAVA S, ZOU Z, PIROLLO K, BLATTNER W, CHANG E. (1990). Germ-line transmission of a mutated p53 gene in a cancer-prone family with Li–Fraumeni syndrome. *Nature*, **348**, 747–749.

STEIN C AND KREIG A. (1994). Problems in the interpretation of data derived from the in vitro and in vivo use of antisense oligonucleotides. *Antisense Res. Dev.*, **4**, 67–69.

SUGIMOTO K, TOYOSHIMA H, SAKAI R, MIYAGAWA K, HAGIWARA K, HIRAI H, ISHIKAWA F AND TAKAKU F. (1991). Mutations of the p53 gene in lymphoid leukemia. *Blood*, **77**, 1153–1156.

THOMAS C. (1992). Regulation of gene expression and function by antisense RNA in bacteria. In *Antisense RNA and DNA*, Murray J (ed.) pp. 51–77. Wiley-Liss: New York.

TOULMÉ, J-J. (1992). Artificial regulation of gene expression by complementary oligonucleotides – an overview. In *Antisense RNA and DNA*, Murray J (ed.) pp. 175–194. Wiley-Liss: New York.

VOJTESEK B, FISHER C, BARNES DM AND LANE DP. (1993). Comparison between p53 staining in tissue sections and p53 proteins levels measured by an ELISA technique. *Br. J. Cancer*, **67**, 1254–1258.

WYLLIE FS, LEMOINE NR, BARTON CM, DAWSON T, BOND J, WYNFORD-THOMAS D. (1993). Direct growth stimulation of normal human epithelial cells by mutant p53. *Mol. Carcinogen.*, **7**, 83–88.